# Supplementary material for: Design and Anticancer Properties of New Water-Soluble Ruthenium–Cyclopentadienyl Complexes
Source: Pharmaceuticals (Basel). 2022 Jul 14;15(7):862. doi: 10.3390/ph15070862 (PMC9321894; doi:10.3390/ph15070862)
Supplement: Supplementary file 1 [file pharmaceuticals-15-00862-s001.zip › pharmaceuticals-1805565-supplementary.pdf]

Design and Anticancer Properties of New Water-Soluble Ruthenium–Cyclopentadienyl Complexes

Tânia S. Morais [1,*], Fernanda Marques [2], Paulo J. Amorim Madeira [3], Maria Paula Robalo [4] and Maria Helena Garcia [1,*]

[1] Centro de Química Estrutural, Institute of Molecular Sciences, Faculdade de Ciências,
Universidade de Lisboa, Campo Grande, 1749-016 Lisboa, Portugal
[2] Centro de Ciências e Tecnologias Nucleares, Instituto Superior Técnico, Universidade de Lisboa,
Estrada Nacional 10 (km 139,7), 2695-066 Lisboa, Portugal; fmarujo@ctn.tecnico.ulisboa.pt
[3] Ascenza Agro, SA, Av. do Rio Tejo-Parq. Ind. Sapec Bay, 2910-440 Setúbal, Portugal; paulo.madeira@ascenza.rovensa.com
[4] Centro de Química Estrutural, Institute of Molecular Sciences, Instituto Superior Técnico,
Universidade de Lisboa, Av. Rovisco Pais, 1049-001 Lisboa, Portugal; mprobalo@deq.isel.ipl.pt

Electrochemical characterization of complexes

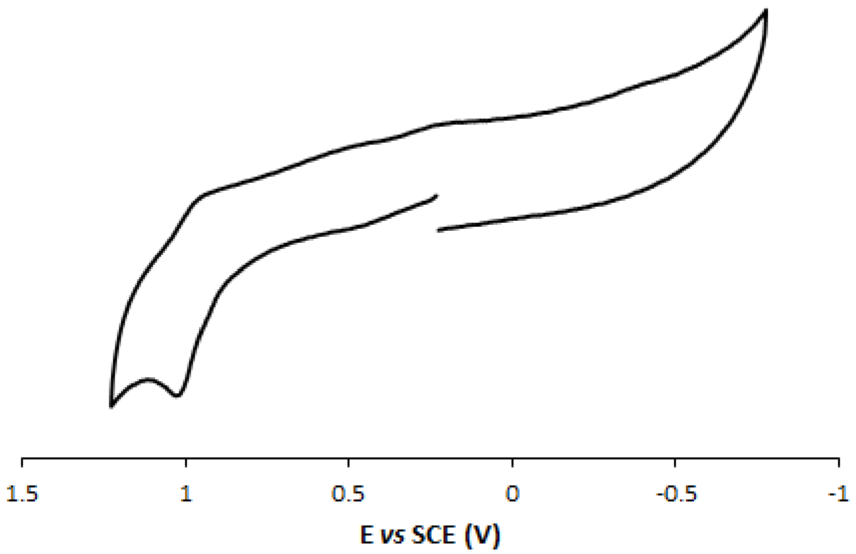

Figure S1. Cyclic voltammogram of complex **7** in dichloromethane at 200 mV.s-1 with a Pt disk WE.

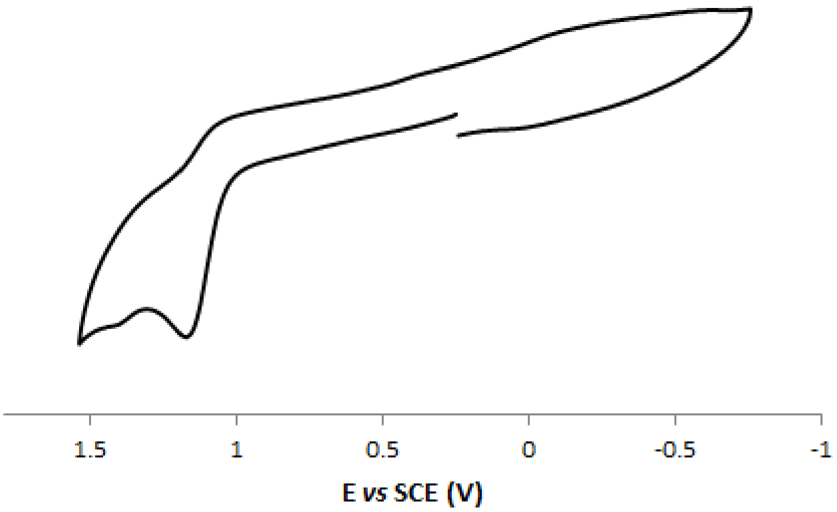

Figure S2. Cyclic voltammogram of complex **8** in dichloromethane at 200 mV.s-1 with a Pt disk WE.

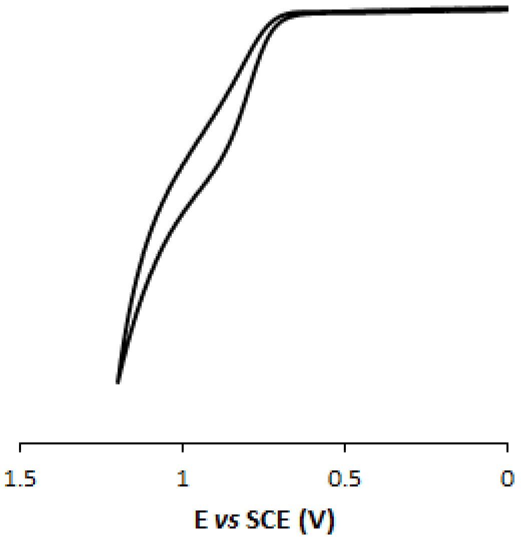

Figure S3. Cyclic voltammogram of complex **1** in Hepes buffer at 50 mV.s-1 with a GC disk WE.

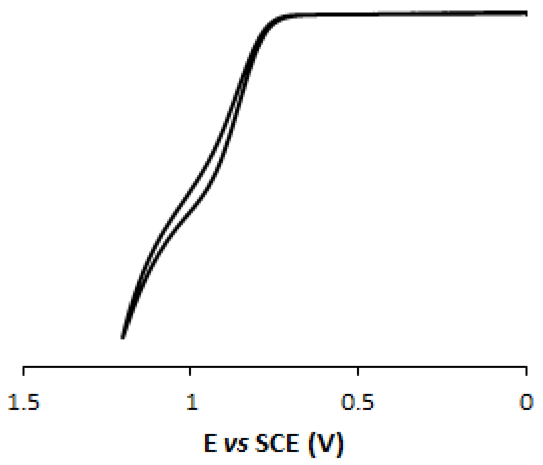

Figure S4. Cyclic voltammogram of complex **2** in Hepes buffer at 50 mV.s-1 with a GC disk WE.

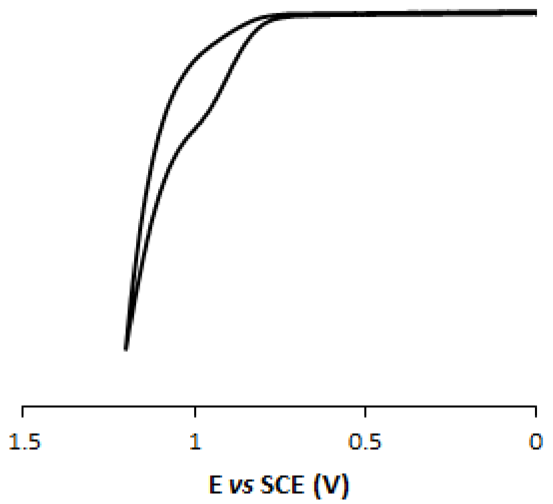

Figure S5. Cyclic voltammogram of complex **3** in Hepes buffer at 50 mV.s-1 with a GC disk WE.

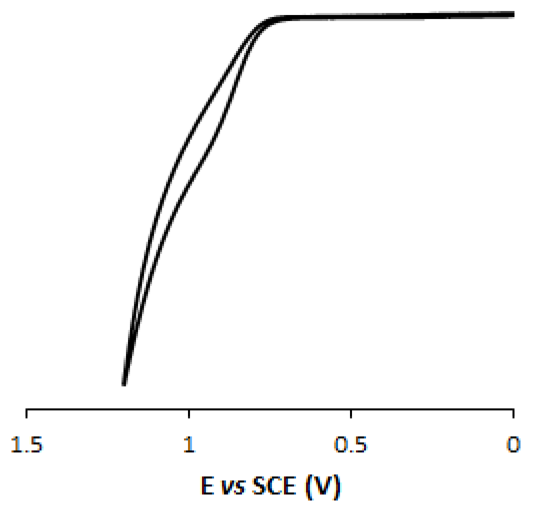

Figure S6. Cyclic voltammogram of complex **4** in Hepes buffer at 50 mV.s-1 with a GC disk WE.

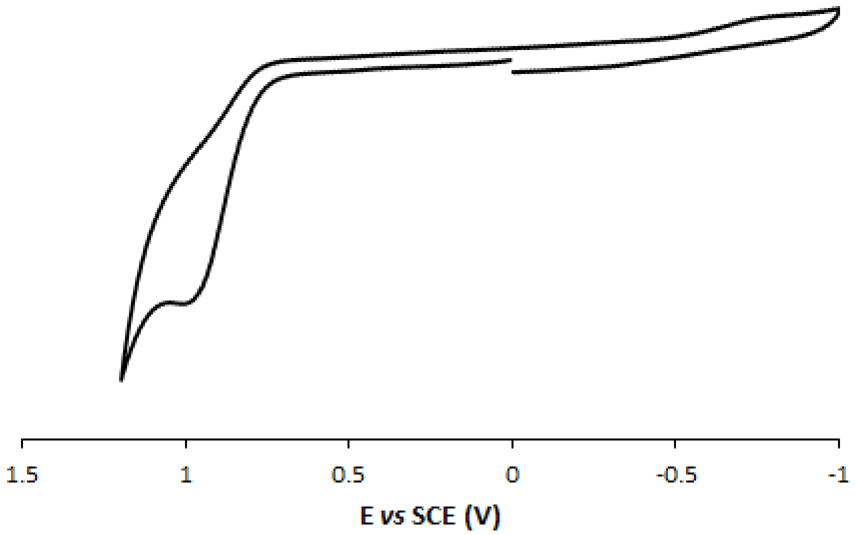

Figure S7. Cyclic voltammogram of complex **5** in Hepes buffer at 50 mV.s-1 with a GC disk WE.

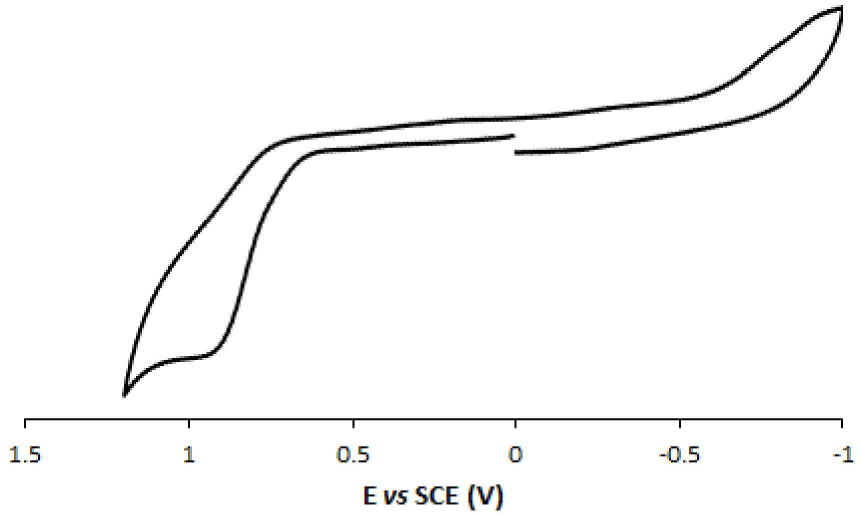

Figure S8. Cyclic voltammogram of complex **7** in Hepes buffer at 50 mV.s-1 with a GC disk WE.

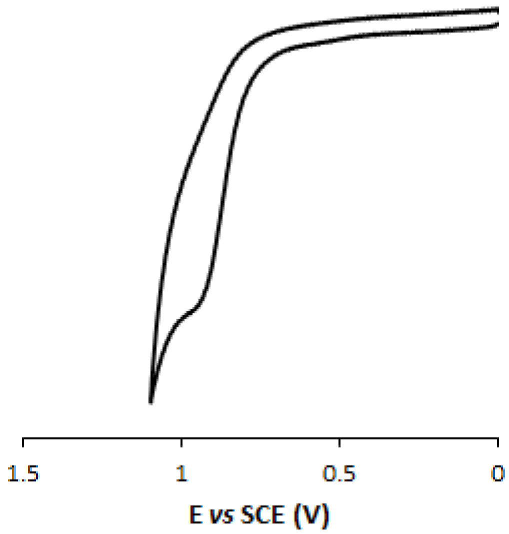

Figure S9. Cyclic voltammogram of complex **8** in Hepes buffer at 50 mV.s-1 with a GC disk WE.

Fluorescence quenching of HSA by complex **[Ru(η^5-C$_5$H$_5$)(*m*TPPMS)(bopy)][CF$_3$SO$_3$] (*6*)**

Stern-Volmer equation:

$$\frac{F_0}{F} = 1 + K_q \tau_0(Q) = 1 + K_{SV}(Q) \qquad (S1)$$

where F_0 and F are the fluorescence intensities of HSA in the absence and presence of the quencher [Ru(η^5-C$_5$H$_5$)(*m*TPPMS)(bopy)][CF$_3$SO$_3$]; [Q] is the quencher concentration; K_q is the bimolecular quenching constant; t_0 is the average fluorescence lifetime of HSA in the absence of the quencher, and K_{SV} is the Stern-Volmer quenching constant.

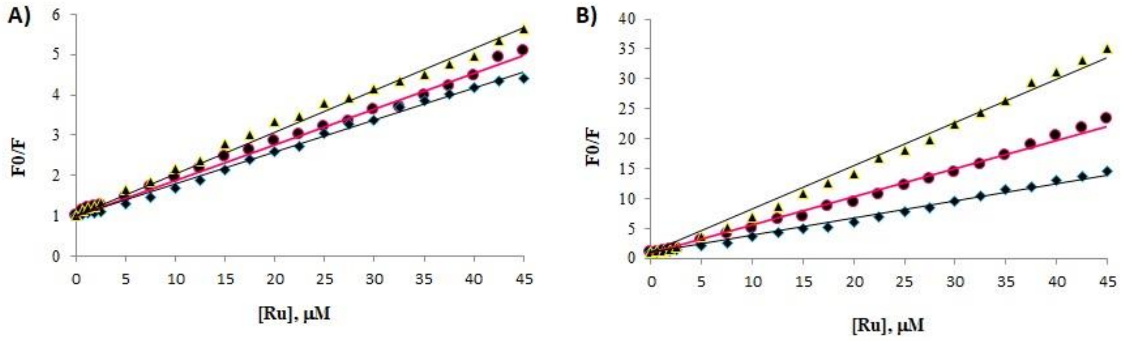

Figure S10. Stern-Volmer plots at pH7.4 for the quenching of (A) HSA and (B) HSA^{faf} by [Ru(η^5-C$_5$H$_5$)(*m*TPPMS)(bopy)][CF$_3$SO$_3$] at three temperatures (◆) 293 K, (●) 298 K, (▲) 310 K.

$$log\left(\frac{F_0 - F}{F}\right) = \log K_a + nlog[Q] \qquad (S2)$$

where K_a is the association constant for a site and n is the number of binding sites by protein.

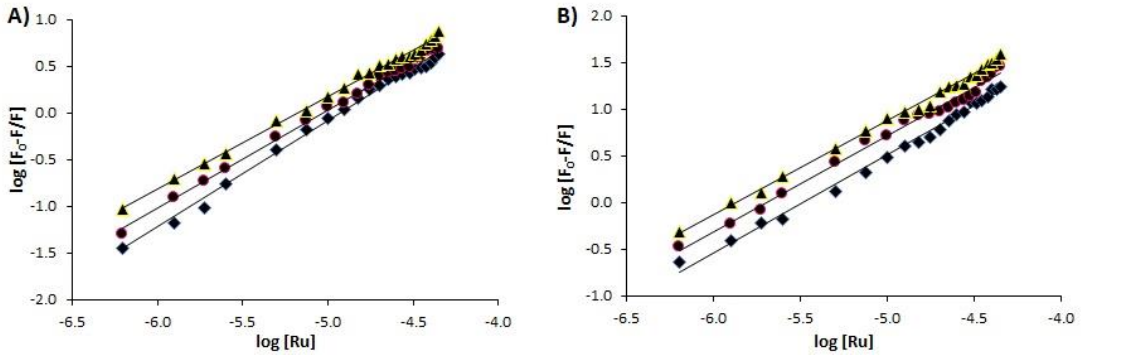

Figure S11. Double-log plots for the fluorescence quenching for the (A) {HSA-[Ru(η^5-C$_5$H$_5$)(*m*TPPMS)(bopy)][CF$_3$SO$_3$]} and (B) {HSA^{faf}-[Ru(η^5-C$_5$H$_5$)(*m*TPPMS)(bopy)][CF$_3$SO$_3$]} systems at three temperatures (◆) 293 K, (●) 298 K, (▲) 310 K.

Van't Hoff equations:

$$lnK_q = -\frac{\Delta H}{RT} + \frac{\Delta S}{R} \qquad (S3)$$

$$\Delta G = \Delta H - T\Delta S \qquad (S4)$$

where R is the gas constant and T is the experimental temperature.

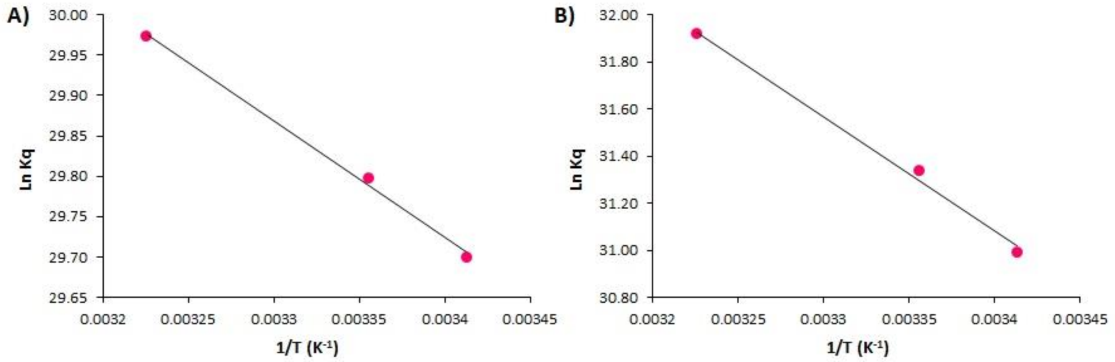

Figure S12. Van't Hoff plots quenching for the (A) {HSA-[Ru(η^5-C_5H_5)(*m*TPPMS)(bopy)][CF_3SO_3]} and (B) {HSA^{faf}-[Ru(η^5-C_5H_5)(*m*TPPMS)(bopy)][CF_3SO_3]} systems.
